# Multiagent therapy with pomalidomide, bortezomib, doxorubicin, dexamethasone, and daratumumab (“Pom‐PAD‐Dara”) in relapsed/refractory multiple myeloma

**DOI:** 10.1002/cam4.3209

**Published:** 2020-07-01

**Authors:** Xiang Zhou, Maximilian J. Steinhardt, Denise Grathwohl, Katharina Meckel, Katharina Nickel, Hans‐Benno Leicht, Franziska Krummenast, Hermann Einsele, Leo Rasche, Klaus M. Kortüm

**Affiliations:** ^1^ Department of Internal Medicine II University Hospital of Würzburg Würzburg Germany

**Keywords:** multiple myeloma, Pom‐PAD‐Dara, refractory, relapse

## Abstract

**Background:**

Even in the era of novel immunotherapies for multiple myeloma (MM), treatment of late‐stage relapsed/refractory (RR) patients remains challenging. The aim of our study was to analyze the efficacy and safety of the five‐drug combination pomalidomide, bortezomib, doxorubicin, dexamethasone, and daratumumab (“Pom‐PAD‐Dara”) in RRMM.

**Methods:**

We retrospectively analyzed data of 56 patients with RRMM who received Pom‐PAD‐Dara between September 2016 and May 2019.

**Results:**

Patients were heavily pretreated with a median of four prior lines of therapy, including autologous and allogenic stem cell transplant in 50 (89%) and six (11%) patients, respectively. The overall response rate (ORR) was 78% and we observed partial remission, very good partial remission, and complete remission in 27 (48%), 13 (23%) and four (7%) patients, respectively. Median progression‐free survival was 7 months (95% CI, 3.3‐10.7) and the median overall survival was not reached at 24 months. Adverse events grade ≥ 3 were observed 41 (73%) patients and included neutropenia (n = 28, 50%), anemia (n = 22, 39%), thrombocytopenia (n = 21, 38%), and pneumonia (n = 6, 11%).

**Conclusion:**

Pom‐PAD‐Dara represents a promising multiagent regimen in heavily pretreated RRMM patients with high ORR and an acceptable safety profile.

## Graphical abstract

We report on our single‐center experience with a novel five drug combination pomalidomide, bortezomib, doxorubicin, dexamethasone, and daratumumab (“Pom‐PAD‐Dara”). The retrospective analysis of 56 patients showed an impressive overall response rate of 78%, including penta‐refractory patients.

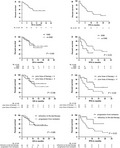

## 1 INTRODUCTION

Despite a growing number of approved drugs, disease relapse is frequently observed in multiple myeloma (MM) and the treatment of late stage relapsed/refractory (RR) disease remains challenging. Novel immunotherapies, such as antibody‐drug‐conjugates, bispecific antibodies, and chimeric antigen receptor T‐cell, may change our future treatment strategies. However, to date, access to these treatments is restricted to a small number of patients within early clinical trials.

Thus, for patients progressing on standard therapy, further treatment options using already approved drugs are urgently needed, as salvage or bridging therapy. This is in particular true for patients that progressed on lenalidomide, pomalidomide, bortezomib, carfilzomib, and daratumumab, the so called “penta‐refractory” disease. For this patient cohort, no effective regimen has yet been established and we report in this manuscript on our single‐center experience with the five‐drug combination pomalidomide, bortezomib, doxorubicin, dexamethasone, and daratumumab (“Pom‐PAD‐Dara”). The aim of this study was to analyze the efficacy and safety of this regimen in patients with RRMM.

## 2 METHODS

### 2.1 Patients

We performed a retrospective single‐center analysis of patients with RRMM who received Pom‐PAD‐Dara in the course of the disease. All procedures followed were in accordance with the ethical standards of the responsible committee on human experimentation (institutional and national) and with the Helsinki Declaration of 1975, as revised in 2008. Informed consent was obtained from all patients for being treated with Pom‐PAD‐Dara and included in the analysis.

Searching our electronic database, we identified and retrieved data of patients who were treated with Pom‐PAD‐Dara from September 2016 to May 2019 at our hospital. MM was diagnosed according to the recent diagnostic criteria recommended by the International Myeloma Working Group (IMWG).^1^ RRMM was defined as per consensus recommendations.^2^ According to the current guidelines, we counted the lines of therapy prior to initiation of Pom‐PAD‐Dara.^3^ Patients were grouped according to the interphase fluorescent in situ hybridization (iFISH) testing of bone marrow plasma cells: high‐risk [del(17p), t(4;14), t(14;16), and t(14;20)] and standard risk (none of all the above) cytogenetics.^4^, ^5^, ^6^ Patients' characteristics at diagnosis of MM and initiation of Pom‐PAD‐Dara, MM‐related data (time point of diagnosis of MM, subtype, cytogenetics, therapy prior to initiation of Pom‐PAD‐Dara, response status at initiation of Pom‐PAD‐Dara and last follow up, survival outcome), and adverse events (AEs) during chemotherapy were collected and evaluated.

### 2.2 Therapy

The Pom‐PAD‐Dara regimen consisted of pomalidomide 4 mg qd orally at bedtime on Day 1‐14; bortezomib 1.3 mg/m^2^ body surface area (BSA) on Day 1, 4, 8, 11 as subcutaneous injection; doxorubicin 9 mg/m^2^ BSA as continuous 24‐hour intravenous (IV) infusion via a central venous access on Day 1‐4; dexamethasone 20 mg qd orally on Day 1, 2, 4, 5, 8, 9, 11, 12; daratumumab 16 mg/kg body weight as IV infusion on Day 0, 5. (Table 1). All the IV infusion therapies were given at the inpatient department. The chemotherapy cycle should be repeated on Day 22 after the evaluation of response. This regimen was modified by the treating physician in case of AEs or anticipated toxicity.

**TABLE 1:** Dosing, dose reduction, and modification in the regimen

Agent	
Bortezomib, n (%)	
1.3 mg/m^2^ on day 1, 4, 8, 11	31 (55)
1.0 mg/m^2^ on day 1, 4, 8, 11	3 (5)
1.3 mg/m^2^ on day 1, 4	12 (21)
1.0 mg/m^2^ on day 1, 4	7 (13)
0.7 mg/m^2^ on day 1, 4	2 (4)
Withdraw after four cycles	1 (2)
Doxorubicin, n (%)	
9 mg/m^2^ on day 1‐4	28 (50)
6 mg/m^2^ on day 1‐4	5 (9)
4 mg/m^2^ on day 1‐4	11 (19)
3 mg/m^2^ on day 1‐4	7 (12)
9 mg/m^2^ on day 1, 4 as bolus	1 (2)
Liposomal doxorubicin 20 mg/m^2^ on day 2, 3	1 (2)
Liposomal doxorubicin 10 mg/m^2^ on day 2, 3	1 (2)
Liposomal doxorubicin 5 mg/m^2^ on day 2, 3	1 (2)
Withdraw and switch to cyclophosphamide 200 mg/m^2^ on day 2, 3	1 (2)
Pomalidomide, n (%)	
4 mg on day 1‐14	31 (55)
3 mg on day 1‐14	3 (5)
2 mg on day 1‐14	22 (40)
Daratumumab, n (%)	
16 mg/kg on day 0, 5	56 (100)
Dexamethasone, n (%)	
20 mg on day 0, 1, 2, 4, 5, 8, 9, 11, 12	55 (98)
Prednisone 50 mg on day 1‐5	1 (2)

All patients received steroid, acetaminophen, and two antihistamines (ranitidine, clemastine) prior to daratumumab. Supportive therapies including acyclovir (400 mg qid), co‐trimoxazole (960 mg qod), pantoprazole (40 mg qd), and thromboprophylaxis with low‐molecular weight heparin (eg, enoxaparin 40 mg qd) or aspirin (100 mg qd) were given in the patients. The treatment with hematopoietic growth factors and transfusion of erythrocytes or thrombocytes were depended on patients' condition and laboratory examination. Pom‐PAD‐Dara was discontinued if the patient did not tolerate it or suffered from disease progression during the treatment.

### 2.3 Response and outcome

The primary objective was the response to Pom‐PAD‐Dara. The secondary end points were the survival outcome and toxicity of the therapy. After each cycle, the response was evaluated according to the current recommendation of IMWG.^7^ Overall response rate (ORR) was defined as the proportion of patients who achieved partial remission (PR) or better. Overall survival (OS) was defined as the period in months from initiation of Pom‐PAD‐Dara to death or the last follow‐up at our institution. Progression‐free survival (PFS) was defined as the interval in months between initiation of Pom‐PAD‐Dara and relapse or progression or, if no relapse or progression occurred, as the time to the last follow‐up. AEs were graded using the Common Terminology Criteria for Adverse Events (CTCAE) Version 4.0.

### 2.4 Statistical analysis

For descriptive statistics data are given as absolute numbers and percentage, and if not otherwise stated as median and minimum and maximum. The survival analysis was performed using Kaplan‐Meier method. We used log‐rank test to compare the survival outcome between subgroups. These analyses were performed with GraphPad Prism 5.0 (GraphPad Software Inc, San Diego, CA, USA). Multivariate analysis using cox regression model was performed with SPSS Statistics 17.0 (SPSS Inc, Chicago, IL, USA). A *P*‐value less than .05 was considered as statistically significant.

## 3 RESULTS

### 3.1 Patients' characteristics

Overall, we identified 56 patients who received Pom‐PAD‐Dara for RRMM. The median age at initiation of Pom‐PAD‐Dara was 61 (range 43‐81) years and the majority of the patients were male (n = 43, 77%). The median interval between the first diagnosis of MM and initiation of Pom‐PAD‐Dara was 48.5 (range 12‐256) months. High‐risk cytogenetics were present in 19 patients (34%). In the subgroup of patients with high‐risk cytogenetics, the median duration between the first diagnosis of MM and Pom‐PAD‐Dara was 33 (range 12‐101) months. Serologic, radiographic, and extramedullary relapse/progression was present in 52 (93%), 25 (45%), and 18 (32%) of patients, respectively. Eight (14%) patients suffered from true extramedullary disease (EMD), and 10 (18%) patients had paramedullary lesions. Fifty‐one (91%) patients had an estimated glomerular filtration rate (eGFR) of ≥30 mL/min. One (2%) patient was dialysis dependent, and this patient received doxorubicin 9 mg/m^2^ on day 1 and 4 as bolus.

The patients were heavily pretreated with a median of 4 (range 1‐10) prior lines of therapy including autologous and allogenic stem cell transplant (SCT) in 50 (89%) and six (11%) patients, respectively. Thirteen (23%), 36 (64%), and one (2%) patients received once, twice, and three times autologous SCT, respectively. Five (9%) patients underwent autologous and allogenic SCT. Five (9%) patients received no SCT in the course of the disease. All the patients were exposed to at least one PI or at least one IMiD, glucocorticoids, and at least one alkylating agent. Twenty‐eight (50%) patients received monoclonal antibodies in the prior therapies. Doxorubicin was given in 41 (73%) patients prior to Pom‐PAD‐Dara. The majority of the patients (n = 42, 75%) was refractory to the last line of therapy. Seven (13%), eight (14%), and 10 (18%) patients were bortezomib‐, daratumumab‐, and pomalidomide‐refractory, respectively. 10 (18%) of the patients were penta‐refractory (resistant to daratumumab, pomalidomide, lenalidomide, carfilzomib, and bortezomib). Patients' characteristics are summarized in Table 2.

**TABLE 2:** Patients' characteristics

Parameter	Missing values, n (%)	
Patients, n		56
Gender, n (%)		
Male		43 (77)
Female		13 (23)
Age at diagnosis of MM, median, years (range)		57 (41‐75)
Subtype, n (%)		
IgG		32 (57)
Non‐IgG		11 (20)
C		13 (23)
Bone marrow infiltration at diagnosis of MM	13 (23)	
Median, % (range)		45 (10‐95)
EMD at diagnosis of MM, n (%)		6 (11)
ISS Stage, n (%)	14 (25)	
I		21 (38)
II		9 (16)
III		12 (21)
Cytogenetics, n (%)	6 (11)	
High‐risk		19 (34)
Standard‐risk		31 (55)
Age at start of Pom‐PAD‐Dara, median, years (range)		61 (43‐81)
Bone marrow infiltration at start of Pom‐PAD‐Dara	28 (50)	
Median, % (range)		50 (0‐100)
eGFR, mL/min (CKD‐EPI)		
Median (range)		69 (16‐120)
Relapse pattern at start of Pom‐PAD‐Dara, n (%)		
Serology		52 (93)
EMD		18 (32)
Bone marrow		10 (18)
Imaging		25 (45)
Elevated lactate dehydrogenase at start of Pom‐PAD‐Dara, n (%)	
Yes		18 (32)
No		38 (68)
Prior lines of therapy, n (%)		
1‐2		13 (23)
3‐5		25 (45)
≥6		18 (32)
Response status at start of Pom‐PAD‐Dara, n (%)		
Refractory to the last line of therapy		42 (75)
Progression from remission		14 (25)
Penta‐refractory		10 (18)
Prior treatment, n (%)		
IMiDs		
Pomalidomide		22 (39)
Lenalidomide		54 (96)
Thalidomide		10 (18)
PIs		
Bortezomib		54 (96)
Carfilzomib		30 (54)
Doxorubicin		41 (73)
Monoclonal antibodies		
Daratumumab		24 (43)
Elotuzumab		9 (16)
SCT		
Prior autologous SCT		50 (89)
Prior allogenic SCT		6 (11)
Best hematological response during Pom‐PAD‐Dara, n (%)		
sCR		2 (4)
CR		2 (4)
VGPR		13 (23)
PR		27 (48)
SD/PD		11 (20)
NA		1 (2)
Overall response rate (≥PR), n (%)		
Refractory to the last line of therapy (n = 43)		32 (74)
Progression from remission (n = 13)		12 (92)
Penta‐refractory (n = 10)		9 (90)

Abbreviations: CR, complete remission; eGFR, estimated glomerular filtration rate; EMD, extramedullary disease; IMiDs, immunomodulatory drugs; ISS, The Multiple Myeloma International Staging System; LC, light chain; M, multiple myeloma; NA, not available; PD, progressive disease; PIs, proteasome inhibitors; Pom‐PAD‐Dara, pomalidomide, bortezomib, doxorubicin, dexamethasone, daratumumab; PR, partial remission; sCR, stringent complete response; SCT, stem cell transplant; SD, stable disease; VGPR, very good partial remission.

### 3.2 Treatment and response to therapy

Patients received a median of 3 (range 1‐14) cycles of Pom‐PAD‐Dara, and 20 (36%) patients obtained five or more cycles. Bortezomib was dose reduced in 24 (43%) patients. In one (2%) patient bortezomib had to be withdrawn due to grade 3 peripheral polyneuropathy after 4 cycles of Pom‐PAD‐Dara. Doxorubicin was given in reduced dose in 24 (43%) patients. Instead of doxorubicin, liposomal doxorubicin was given on day 2, 3 in three (5%) patients at a dose of 5, 10, and 20 mg/m^2^ BSA, respectively; these three patients had received doxorubicin containing prior therapy and reached a cumulative dose of >450 mg/m^2^ and we observed no sign of heart failure. In one (2%) patient with concomitant cardiac amyloidosis, we had to replace doxorubicin with cyclophosphamide due to worsening of cardiac function. This patient achieved a serologic VGPR and cardiac response with >30% reduction in NT‐proBNP according to the current response criteria.^8^ Pomalidomide was dose reduced in 25 (45%) patients. In one (2%) 81‐year‐old patient, we replaced dexamethasone with prednisone at a dose of 50 mg qd on day 1‐5. Overall, dose of any of the agents was reduced in 39 (70%) patients. In 15 (27%) and 24 (43%) patients, dose was reduced due to toxicity of Pom‐PAD‐Dara or preexisting condition, respectively. Dosing and dose reduction are summarized in Table 1.

The best response to Pom‐PAD‐Dara was available in 55 (98%) patients. One (2%) patient died during the first cycle of Pom‐PAD‐Dara due to sepsis. Therefore, it was presumably a toxic death, and the hematological response status was not available. The ORR was 78% and we observed PR, very good partial remission (VGPR) and complete remission in 27 (48%), 13 (23%), and four (7%) patients, respectively. Two (4%) of the four patients achieved a stringent complete response and minimal residual disease negativity at a sensitivity level of 10^−4^ and 10^−6^, respectively. In the entire cohort, 11 (20%) patients did not respond to Pom‐PAD‐Dara. Among the 10 patients with penta‐refractory MM, four and five patients achieved VGPR and PR, respectively, and one (2%) patient did not respond to Pom‐PAD‐Dara.

### 3.3 Survival analyses

**FIGURE 1:**
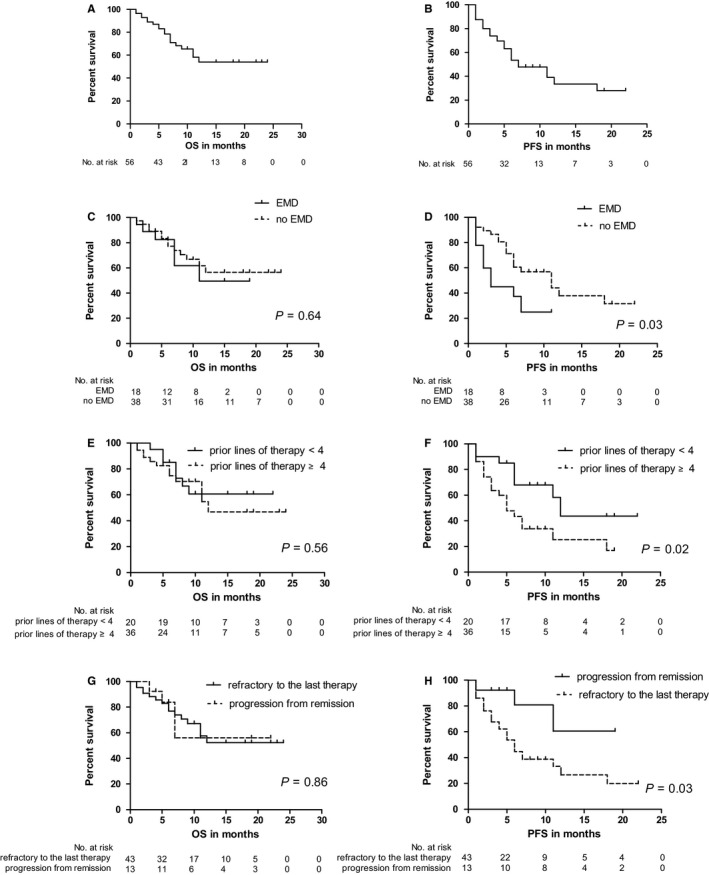
Overall survival (OS) (A) and progression‐free survival (PFS) (B) of the entire group (n = 56); OS (C) and PFS (D) in patients with and without extramedullary disease (EMD) (with EMD, n = 18; without EMD, n = 38); OS (E) and PFS (F) with regard to prior lines of therapy (≥4, n = 36; <4, n = 20); OS (G) and PFS (H) of patients who were refractory to the last line of therapy (n = 43) and who were not (n = 13)

Overall, the median OS was not reached at 24 months, and the median PFS was seven months (95% CI, 3.3‐10.7) (Figure 1A,B). Patients with EMD at relapse had a significantly inferior PFS compared with patients without EMD (*P* = .03), and there was no difference in OS between the both groups (*P* = .64) (Figure 1C,D). Moreover, we observed a significantly superior PFS in patients who received less than four lines of prior therapy compared with those with four or more prior lines of therapy (*P = *.02, Figure 1F). However, there was no difference in OS between the both groups of patients (*P = *.56, Figure 1E). Furthermore, patients who were refractory to the last line of therapy had a significantly inferior PFS compared with those with a progression from remission (*P = *.03, Figure 1H). However, we observed no difference in OS between the both patient groups (*P = *.86, Figure 1G). In the multivariate analysis using cox regression model considering the three above mentioned factors, however, none of them had a significant influence on PFS.

### 3.4 Adverse events

All the IV infusion therapies were given in the inpatient department of our institution. During the hospital stay, AEs grade ≥ 3 were observed in 41 (73%) patients. Overall, anemia, leukopenia, neutropenia, and thrombocytopenia grade ≥ 3 were observed in 22 (39%), 27 (48%), 28 (50%), and 21 (38%) patients, respectively (Table 3). Preexisting neutropenia, anemia, and thrombocytopenia ≥ grade 3 were observed before initiation of Pom‐PAD‐Dara in nine (16%), eight (14%), and nine (16%) patients, respectively. Patients with leukopenia and/or neutropenia received (pegylated) granulocyte colony stimulating factor (G‐CSF) until the recovery of leukocyte and neutrophil granulocyte. Pneumonia (n = 6, 11%) was the most common nonhematologic AE grade ≥ 3 (Table 3). One patient died during the chemotherapy due to respiratory syncytial virus (RSV) pneumonia. Another patient with extramedullary relapse and subtotal bone marrow infiltration died during the first cycle of Pom‐PAD‐Dara due to sepsis.

**TABLE 3:** Adverse events during hospital stay

	Any grade ≥ 2	Grade 3	Grade 4
Hematologic events, n (%)		
Anemia	41 (73)	22 (39)	
White blood cell decreased	44 (79)	17 (30)	10 (18)
Neutrophil count decreased	40 (71)	13 (23)	15 (27)
Platelet count decreased	25 (45)	9 (16)	12 (21)
Febrile neutropenia	2 (4)	2 (4)	
Nonhematologic events, n (%)		
Pneumonia	7 (13)	6 (11)	
Bronchospasm	2 (4)	1 (2)	
Lymph node infection	1 (2)	1 (2)	
Liver enzyme increased	3 (5)	3 (5)	
Enterocolitis infectious	1 (2)	1 (2)	
Catheter‐related infection	2 (4)	1 (2)	
Peripheral polyneuropathy	1 (2)	1 (2)	
Heart failure	2 (4)	2 (4)	
Thromboembolic events	2 (4)		
Diarrhea	1 (2)		
Flu‐like symptoms	3 (6)		
Agitation	1 (2)		
Stroke	1 (2)		
Atrial fibrillation	1 (2)		
Death	2 (4)		

## 4 DISCUSSION

In this study, we retrospectively analyzed the data of patients who received Pom‐PAD‐Dara as salvage therapy in the RRMM setting. Overall, we observed an ORR of 78% in a cohort of patients who were heavily pretreated with a median of four (range 1‐10) prior lines of therapy.

Bortezomib, doxorubicin, and dexamethasone (PAD) is an established regimen in patients with RRMM that has been demonstrated in diverse previous studies.^9^, ^10^, ^11^ Moreover, intensive chemotherapy using VTD‐PACE‐like regimen (bortezomib, thalidomide, dexamethasone, cisplatin, doxorubicin, cyclophosphamide, and etoposide) has been shown to be effective in patients with RRMM.^12^ Furthermore, for patients with extramedullary advanced MM, Dexa‐BEAM‐like regimen (dexamethasone, carmustine [BCNU], etoposide, cytarabine, and melphalan) is an effective induction prior to an intended autologous or allogenic SCT.^13^ Recently, pomalidomide and monoclonal antibodies based regimens have shown promising efficacy in patients with RRMM. For instance, Dimopoulos et al reported that patients with lenalidomide refractory RRMM were treated with elotuzumab plus pomalidomide and dexamethasone and achieved an ORR of 53%.^14^ In a study by Chari et al (EQUULEUS trial), an ORR of 60% was observed in patients with RRMM that were treated with daratumumab plus pomalidomide and dexamethasone.^15^ In other studies, evaluating daratumumab plus pomalidomide and dexamethasone, the ORRs were 36.8% and 46%, respectively.^16^, ^17^ Our findings suggested that Pom‐PAD‐Dara improved the ORR compared with already FDA (Food and Drug Administration) approved regimen daratumumab plus pomalidomide and dexamethasone. In this context, the updated analysis of CASTOR trial showed an ORR of 83.8% in the subgroup of daratumumab plus bortezomib and dexamethasone. However, the patients in the CASTOR trial were less heavily pretreated with a median of two (range 1‐9) prior lines of therapy compared with our cohort.^18^ Notably, also patients with penta‐refractory MM responded to Pom‐PAD‐Dara with an ORR of 90%. Our results demonstrated that Pom‐PAD‐Dara might also overcome resistance to single agent pomalidomide, bortezomib, or daratumumab.

In our cohort, the median PFS from initiation of Pom‐PAD‐Dara was 7 months (95% CI, 3.3‐10.7) and the median OS was not reached at 24 months. Similarly, the results of EQUULEUS trial evaluating daratumumab plus pomalidomide and dexamethasone showed a survival outcome with a median PFS of 8.8 months and a median OS of 17.5 months, respectively.^15^ However, Hussain et al and Lakshman et al reported inferior PFS of 3.9 and 5.2 months in patients receiving daratumumab plus pomalidomide and dexamethasone when compared with our cohort.^16^, ^17^ Furthermore, we noticed that resistance to the prior therapy lines indicated an inferior PFS in patients receiving Pom‐PAD‐Dara. Our findings suggested that earlier initiation of Pom‐PAD‐Dara might improve the PFS of patients with RRMM.

In total, AEs grade ≥ 3 were observed in 41 (73%) patients and included neutropenia (n = 28, 50%), anemia (n = 22, 39%), thrombocytopenia (n = 21, 38%), and pneumonia (n = 6, 11%). However, preexisting neutropenia, anemia, and thrombocytopenia ≥ grade 3 were observed in nine (16%), eight (14%), and nine (16%) patients, respectively. Two patients died during Pom‐PAD‐Dara due to RSV pneumonia or sepsis, respectively. Our findings showed a safety profile comparable to that of EQUULEUS (daratumumab, pomalidomide, and dexamethasone) and CASTOR (daratumumab, bortezomib, and dexamethasone) trial.^15^, ^18^ There were several limitations of our study. First, this is a retrospective, single‐center analysis of relatively small number of cases. Second, we could only collect and analyze the data of AEs during hospital stay and data after discharge of patients were not available in our electronic database. Thus, it is likely that more AEs could have occurred during the entire course of Pom‐PAD‐Dara, but these were not high grade and did not require hospitalization. Third, due to the small number of cases the multivariate survival analysis using cox regression model should be interpreted with caution. Fourth, due to the continuous doxorubicin infusion, Pom‐PAD‐Dara requires a hospitalization of at least 4 days per cycle, which led to increased cost of treatment. The issue of health economics represents another hurdle especially for countries with limited medical resources and, therefore, also a limitation of our study.

In conclusion, our findings demonstrated the efficacy with high ORR and the acceptable safety profile of the multiagent regimen Pom‐PAD‐Dara in heavily pretreated RRMM patients. Also patients with penta‐refractory MM could benefit from this treatment.

## CONFLICTS OF INTEREST

All authors declare that they have no conflicts of interest relevant to the submitted manuscript.

## AUTHORS' CONTRIBUTIONS

XZ, KMK, and LR designed the study, analyzed and interpreted the data, and drafted the work; MJS, DG, KM, KN, HBL, FK, and HE conceived the design of the work and substantially revised it. All authors have approved the submitted version.

## Data Availability

Data presented in this study are available in the article or from the corresponding author upon reasonable request.
